# Suicide With Attempted Filicide by Poisoning: A Case Report

**DOI:** 10.7759/cureus.90143

**Published:** 2025-08-15

**Authors:** Toshal Wankhade, Kuldeep Sarmah, Auroprajna Pal, Binay Kumar, Amit Patil, Chaitanya Mittal

**Affiliations:** 1 Forensic Medicine and Toxicology, All India Institute of Medical Sciences, Patna, Patna, IND

**Keywords:** dyadic death, filicide, forensic toxicology, organophosphorus poisoning, suicidal poisoning

## Abstract

Suicide by poisoning, particularly through the ingestion of agricultural pesticides, remains a prevalent method of self-harm in India due to their widespread availability and accessibility. We present a case of a married woman who intentionally ingested an agricultural pesticide and simultaneously administered the same substance to her four children by mixing it into tea. Despite intensive medical intervention, the woman succumbed to poisoning, whereas all four children survived. This case constitutes an unusual instance of suicide associated with attempted filicide by the use of pesticides. Motivating factors for this tragic event included prolonged domestic abuse, financial hardship, and social isolation. The report discusses the clinical course and forensic evaluation, including clinical forensic assessment, autopsy examination, and medicolegal aspects of pesticide poisoning (organophosphorus compound), emphasizing the toxicological profile of such agents. The report also emphasizes the broader public health implications of intentional poisoning events and the forensic significance of filicide associated with maternal suicide.

## Introduction

Filicide, derived from the Latin words filius (son) or filia (daughter) and the suffix -cide meaning “to kill,” refers to the act of a parent or parental figure deliberately causing the death of their child under the age of 18 years [1]. Although globally recognized, filicide is influenced by diverse psychological and sociocultural factors. Historically, it ranged from legal permissibility in Greco-Roman times to modern interpretations shaped by psychiatry and human rights [2]. 

Homicide and suicide instances occurring together are known as dyadic death. Filicide-suicide is one such type of instance where children are victims and parents are perpetrators. Filicide-suicide with parental suicide is particularly under-documented, especially in rural areas where social stigma and lack of awareness obscure both causation and reporting. The motives in such cases range from altruistic intent, where the parents believe they are saving the child from a worse fate, to neglect or distorted protective instincts. In contemporary contexts, maternal filicide is reported more frequently than paternal filicide and is often associated with mental illness, postpartum psychiatric disorders, intimate partner violence, or severe socio-economic hardship [3-5].

Poisoning cases by organophosphorus compounds are mostly suicidal in nature [6]. In India, agricultural poisons are the leading agents in fatal poisoning cases, with suicidal intent being especially prevalent in rural settings. These pesticides are readily available in agricultural households and are highly toxic. Their low cost, high lethality, and lack of regulation make them a common agent for self-harm and impulsive violence. In India, their use is less frequently reported among females, and homicidal applications are rare [7]. In the present case, we encountered a unique scenario in which a woman ingested an agricultural poison in an act of suicide and simultaneously attempted filicide, a combination scarcely documented in various documented homicide-suicide instances [3,5].

This report describes a suicide attempt involving organophosphorus poisoning followed by attempted filicide, highlighting its toxicological, medicolegal, and psychosocial implications.

## Case presentation

This case report is about a woman, along with her four children, who was admitted to our hospital as a case of poisoning. The mother is a 36-year-old married woman residing in a rural region of the northern part of India. She belongs to a family of low socio-economic status and was married for 12 years. She had four children aged between 3 and 10 years (two boys and two girls). Her husband, an unemployed and habitual alcoholic, was known to be physically and verbally abusive. The family faced persistent financial instability.

Neighbors and local community members revealed that, in the weeks leading up to the incident, the woman had exhibited signs of emotional exhaustion and withdrawal and expressed feelings of hopelessness. She had no documented history of psychiatric illness, though it is likely she was experiencing undiagnosed depression or adjustment disorder under chronic stress.

As per the history obtained from the deceased woman’s elder daughter and the police investigation, the woman prepared tea and mixed a liquid pesticide into it, which she had reportedly purchased a week prior. She consumed the tea herself and also served it to her four children, reportedly without their knowledge, as their morning beverage on an empty stomach. Within 30-60 minutes, the woman and her children began showing symptoms of poisoning. A neighbor, hearing the cries of the children and witnessing vomiting, alerted local health workers.

All five were initially brought to a nearby primary health center (PHC), where basic resuscitative measures were promptly initiated. These included the administration of intravenous fluids, atropine bolus injections to counteract cholinergic symptoms, and gastric lavage to reduce further absorption of the ingested poison. Given the mother’s progressively deteriorating condition and the limited resources available at the PHC, all of them were urgently referred to our institute for further toxicological management and advanced supportive care.

Upon admission to our hospital, the mother was found to be restless, disoriented, and gasping for breath. Her vital parameters revealed a respiratory rate of 30/min (labored), blood pressure of 90/70 mmHg, heart rate of 120 bpm (tachycardia), and oxygen saturation (SpO₂) of 92% on room air. Neurologically, she was disoriented with a Glasgow Coma Scale (GCS) score of 10/15. Chest auscultation revealed bilateral basal crepitations, and she exhibited classic cholinergic signs, including profuse salivation, constricted and reactive pupils, bradycardia episodes, and hypotonia. Emergency treatment was initiated with atropine and pralidoxime. Supportive management included oxygen therapy via a non-rebreather mask (NRBM) at 10 L/min, a 500 ml bolus of Dextrose Normal Saline (DNS), and the administration of antiemetics and sedatives. Due to a declining GCS and progressive hypoxia, endotracheal intubation was performed six hours post-admission, and she was placed on mechanical ventilation to maintain SpO₂ above 95%. Despite aggressive resuscitative efforts, her condition deteriorated with the development of respiratory acidosis, pulmonary edema, and refractory hypotension. She died after five days of intensive care, and as per medical records, death occurred due to fatal organophosphorus compound poisoning.

All four children presented with mild to moderate symptoms of poisoning, including nausea, vomiting, and mild diarrhea. They were drowsy but remained hemodynamically stable, with blood pressure and oxygen saturation levels within normal limits without any focal neurological deficits or signs of respiratory compromise. Immediate interventions included gastric lavage followed by continuous infusion of atropine at a dose of 0.02 mg/kg/hour for 28 hours and pralidoxime infusion at 10 mg/kg/hour. All children were admitted to the Pediatric Intensive Care Unit (PICU) for close monitoring. Throughout the course of hospitalization, they were carefully observed for the development of intermediate syndrome, delayed neurotoxicity, or respiratory depression; however, none of these complications were noted. Following clinical improvement and stabilization, the children were discharged after 13 days with referrals for psychiatric evaluation and ongoing social support interventions.

**Figure 1:**
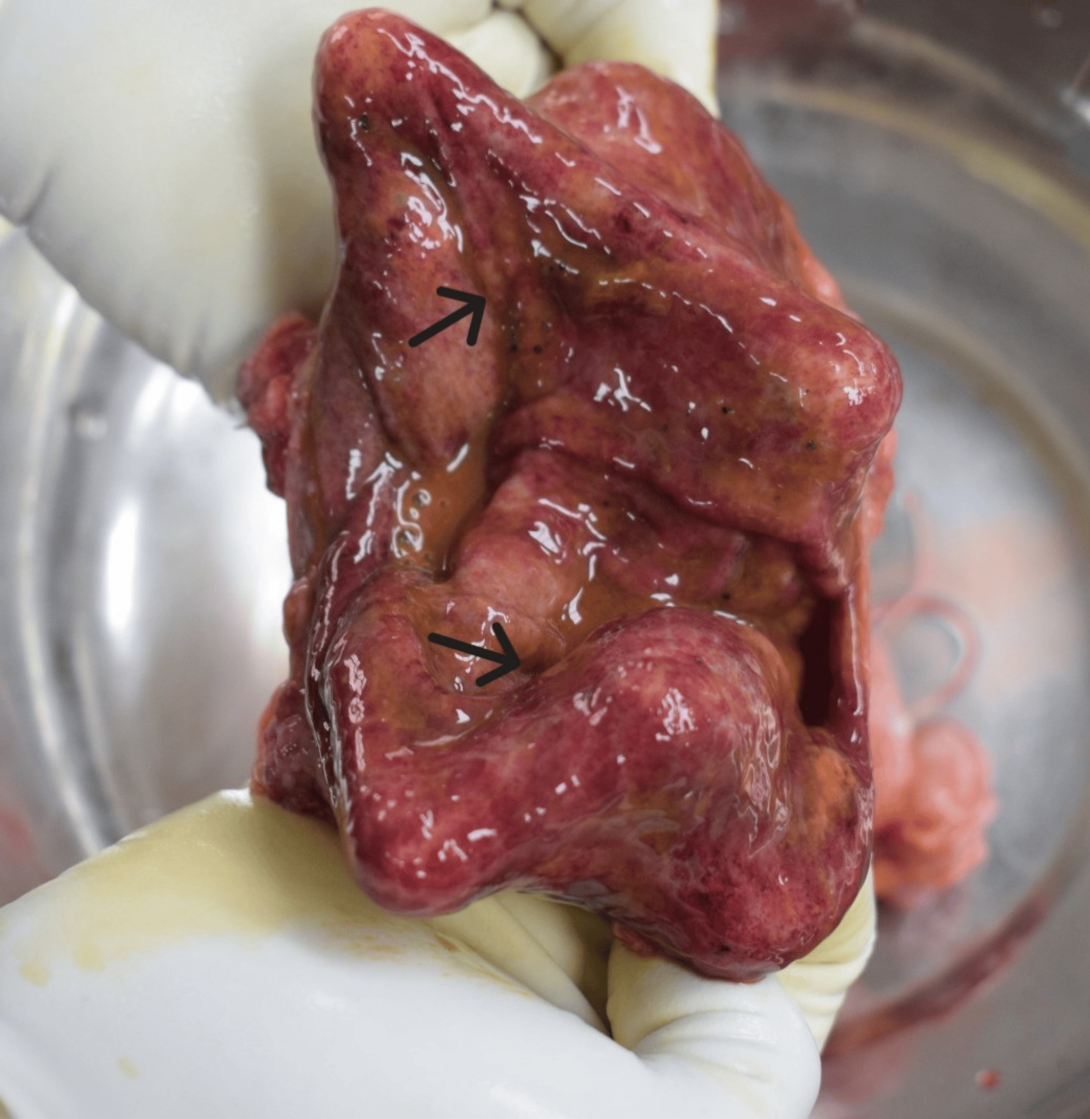
Highly decongested stomach mucosa.

**Figure 2:**
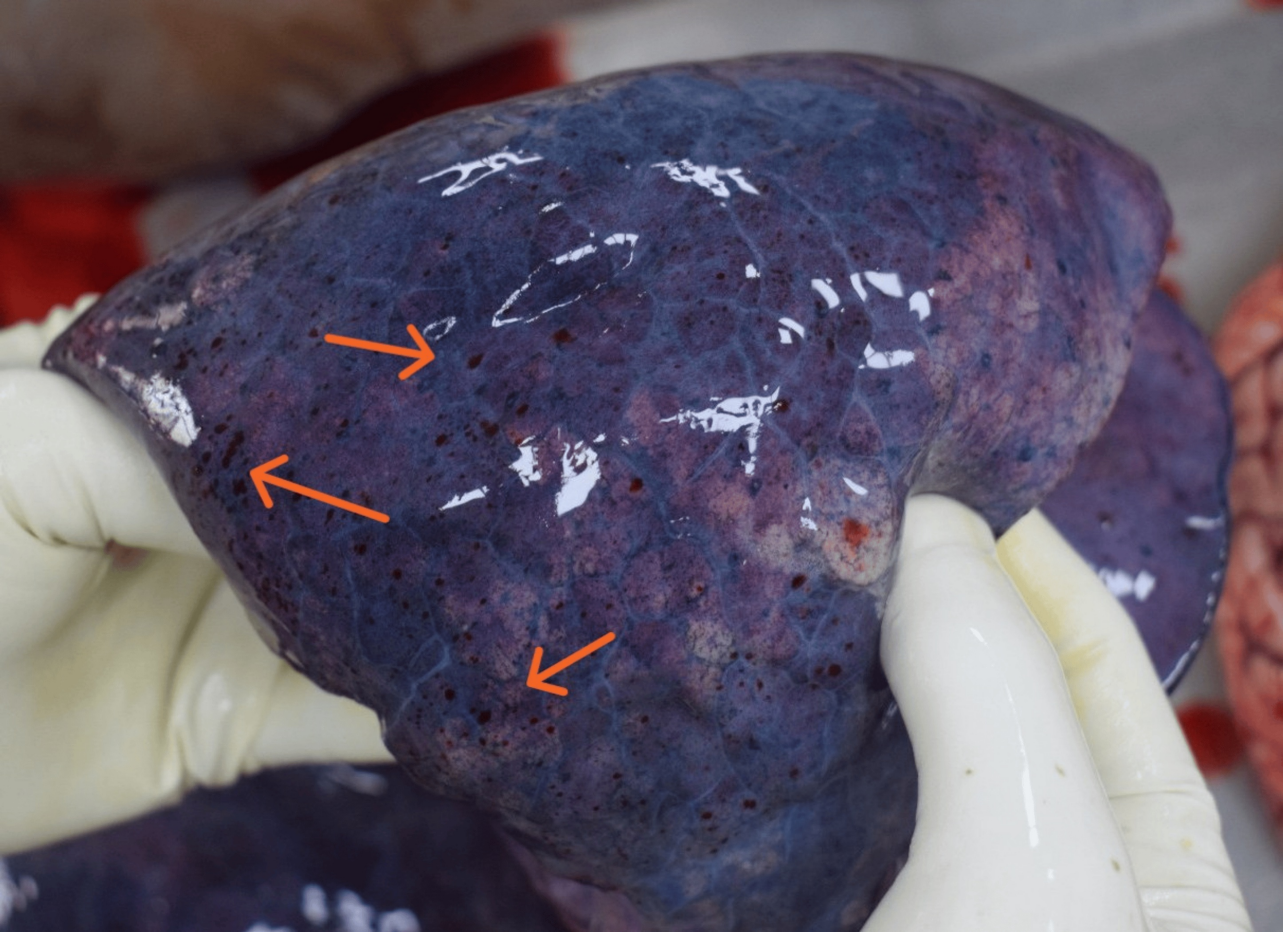
Petechial hemorrhages on the lung.

**Figure 3:**
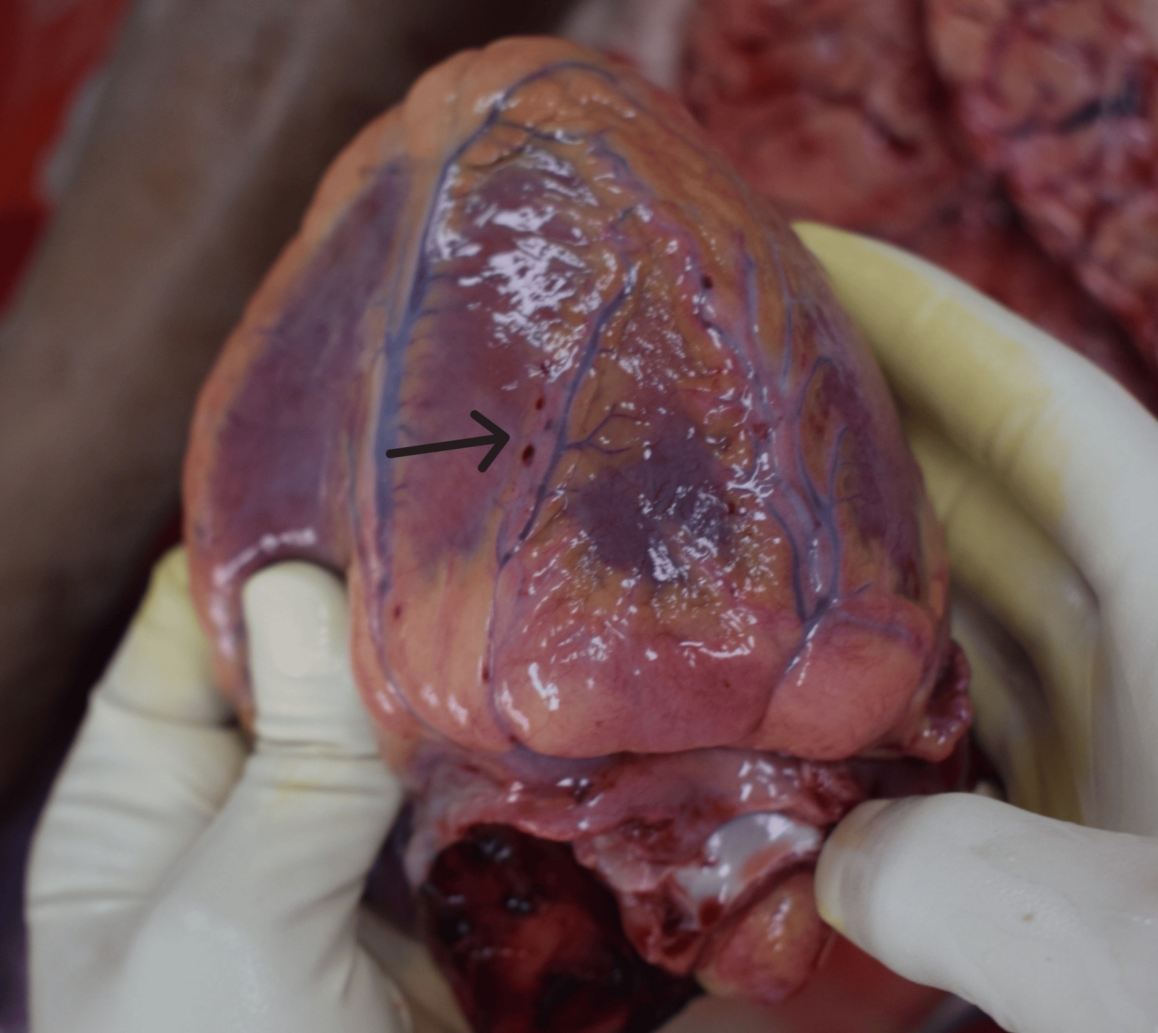
Petechial hemorrhages on the heart.

The postmortem examination of the deceased mother was conducted by our department as per standard medicolegal autopsy protocol. On external examination, cyanosis was noted on the lips, nail beds, and toes, with a slight blood-tinged froth present at the mouth and nostrils. No external mechanical injuries were observed. A strong offensive odor was distinctly detected from the gastric cavity; the stomach mucosa appeared congested with patchy red hemorrhagic spots suggestive of ingestion of a toxic substance (Figure 1). Internal examination revealed an edematous brain with congested meninges; no evidence of intracranial hemorrhages was found. The pleural cavities contained about 500 ml of yellowish fluid on each side. Both lungs were markedly edematous (right: 740 g, left: 620 g) and showed multiple petechial hemorrhages on pleural and parenchymal surfaces (Figure 2). The heart shows petechial hemorrhages on the wall of the left ventricle (Figure 3). All visceral organs were congested. 

Viscera samples, including the stomach and contents, part of the liver, kidney, spleen, and blood sample, were sent for chemical analysis as per protocol. Similarly, the gastric lavage fluid preserved during treatment was also sent to the Forensic Science Laboratory for chemical analysis. Gastric lavage samples of all four children were also forwarded for chemical analysis. The postmortem examination findings of the deceased mother were consistent with death due to poisoning. Furthermore, the clinical presentation and treatment records of all the patients collectively indicated that the poisoning resulted from ingestion of an organophosphorus compound. 

## Discussion

This case represents a tragic yet illustrative example of maternal filicide by use of poison, probably by an organophosphorus compound. Driven by chronic social stressors and executed using a commonly available toxic compound, it reflects the convergence of medical emergency, mental health crisis, and systemic social failure, particularly in low-income, rural Indian households.

Clinical and toxicological considerations

Organophosphorus (OP) compounds are potent neurotoxins that inhibit acetylcholinesterase, leading to the accumulation of acetylcholine and overstimulation of muscarinic and nicotinic receptors. Clinical manifestations typically include salivation, lacrimation, urination, defecation, gastrointestinal distress, emesis, bradycardia, and miosis [8].

In the present case, the mother exhibited classic cholinergic symptoms, requiring immediate resuscitation, atropine therapy, and mechanical ventilation. Despite adequate medical support, her condition deteriorated, suggesting a significantly higher dose exposure. OP poisoning is associated with a high case fatality rate if not treated aggressively within the initial hours.

The children also developed cholinergic features such as nausea, vomiting, abdominal pain, lacrimation, and pupillary constriction. However, they responded well to early initiation of atropine and pralidoxime. Children are generally more sensitive and vulnerable to OP poisoning due to their smaller body size, immature detoxification mechanisms, and a relatively larger absorbed dose per kilogram. Nevertheless, population studies often report better outcomes in children because unintentional exposures typically involve lower doses compared to deliberate ingestions in adults [9,10].

This case highlights the importance of prompt and aggressive clinical management. The mother’s fatal outcome was likely due to intentional high-dose ingestion of poison. Furthermore, as OP compounds have a strong, unpleasant odor, covert administration is difficult, and they do not meet the criteria for an “ideal homicidal poison” [11]. Therefore, the lower ingested dose in the children likely played a crucial role in their survival. However, the exact dose of poison consumed by children was not known, which is a limitation of this report. This inference is based on an assumption.

During the course of treatment, it has been observed that serum lactate levels and low arterial pH act as independent predictors of mortality in acute OP poisoning. A study by W. Tang et al. [12] has identified high serum lactate levels, low arterial pH, and reduced lactate clearance within six hours of admission as independent predictors of mortality in acute OP poisoning. In the present case, all the children and the mother had high lactate levels and metabolic acidosis at the time of admission. With intensive treatment, the biochemical markers improved in all the children but remained continually deranged in the mother. This highlights the importance of biochemical markers in triaging patients and guiding the intensity of therapy.

Forensic challenges in poisoning cases 

From a medico-legal perspective, this case highlights several critical issues. Despite clear clinical manifestations and autopsy findings consistent with poisoning, chemical analysis results may be negative if biological samples are improperly stored, degraded, or submitted with significant delays. Additionally, prolonged hospitalization can further reduce the likelihood of detecting toxins. This phenomenon is known as viscera-negative poisoning [13]. It complicates forensic interpretation and underscores the necessity of integrating clinical observations, circumstantial evidence, postmortem findings, and toxicological reports in such investigations.

In our case, a gastric lavage sample obtained shortly after hospital admission was preserved and promptly submitted for chemical analysis, with immediate police notification. This step is crucial, as early gastric lavage is more likely to yield positive toxicological results compared to viscera samples collected during postmortem examination after extended hospitalization. Therefore, it is essential for legal authorities to recognize that accurate medico-legal conclusions in such cases require a comprehensive approach integrating clinical evidence, circumstantial details, postmortem findings, and toxicological results rather than relying solely on chemical analysis reports.

Psychosocial aspect

Filicide has been extensively studied in psychiatric literature and is almost universally linked with overwhelming psychological distress, untreated mental illness, or socioeconomic despair. The woman in this case had no formal psychiatric diagnosis but lived in an abusive marriage with an alcoholic husband, under financial pressure, and with the responsibility of raising four children alone. According to West et al. and Resnick's classification, maternal filicide can be categorized into several types: altruistic, acutely psychotic, fatal maltreatment, unwanted child, and spousal revenge. The current case aligns with the altruistic subtype, wherein the mother, perceiving her situation as hopeless, believed that death would spare her children from suffering. This study mentions that the incidence of filicide has relatively equal involvement of both males and females [14]. However, a study by Gupta BD [5] suggests more involvement of the mother as the perpetrator.

Research by Sahin et al. revealed that over 67% of organophosphate poisoning victims were female, with a mean age of 30 years. The study also mentions that psychosocial factors and the lack of regulation in the sale of these poisons play a key role in this context [6]. However, in the Indian scenario, the involvement of the male population is higher compared to females in suicidal deaths caused by organophosphorus compounds. Nonetheless, organophosphorus compounds remain one of the most common methods used for suicide [15]. As per data published in India for 2020, about 25% of suicides were due to poisoning, making it the second most common method after hanging, which accounted for roughly 53% of cases. Suicidal hanging was more common among young females, accounting for half of the female fatalities in the third decade, followed by the second decade. This age group may have a higher likelihood of suicide due to the increased stress that a woman is likely to feel before and after marriage [16]. Multiple studies have demonstrated that young, illiterate, and socioeconomically disadvantaged women are disproportionately affected in suicidal poisoning cases, particularly in rural settings. The easy availability of pesticides, coupled with minimal awareness about their lethality, makes them a tool for impulsive self-harm and violence against others [7,16]. 

In India, social stigmas and a lack of mental health access often prevent women from seeking timely support. Combined with patriarchal norms and economic dependency, this creates a toxic environment of learned helplessness. Domestic violence is an established precursor to suicidal ideation in women, and when children are involved, it can manifest as extended suicide or attempted filicide [3]. 

## Conclusions

This case illustrates how socio-economic hardship, domestic violence, and psychosocial pressures can drive vulnerable populations, especially rural women, to extreme acts such as filicide followed by suicide. The contrasting outcomes between the mother and her children highlight the severe and unpredictable nature of organophosphorus poisoning, likely influenced by variations in dose, manner of administration, and pediatric physiological responses. From a forensic perspective, it reinforces the need for meticulous documentation, timely sample preservation, and correlation of all evidence to avoid viscera-negative pitfalls. Ultimately, it underscores the urgent need for stricter regulation of toxic substances, accessible mental health care, proactive domestic violence screening, and community vigilance to protect vulnerable families.
